# Use of MR Urography in Pediatric Patients

**DOI:** 10.1007/s11934-018-0843-7

**Published:** 2018-09-11

**Authors:** Cara E. Morin, Morgan P. McBee, Andrew T. Trout, Pramod P. Reddy, Jonathan R. Dillman

**Affiliations:** 1 Department of Diagnostic Imaging, St. Jude Children’s Research Hospital, 262 Danny Thomas Place, Memphis, TN 38105 USA; 2 Department of Radiology, Medical University of South Carolina, Charleston, SC USA; 3 Department of Radiology, University of Cincinnati College of Medicine, Cincinnati, OH USA; 4 Department of Radiology, Cincinnati Children’s Hospital Medical Center, Cincinnati, OH USA; 5 Division of Pediatric Urology, Cincinnati Children’s Hospital Medical Center, Cincinnati, OH USA

**Keywords:** Imaging, Children, Kidneys, Urinary tract, Hydronephrosis, Renal transplant

## Abstract

**Purpose of Review:**

In this article, we describe the basics of how magnetic resonance urography (MRU) is performed in the pediatric population as well as the common indications and relative performance compared to standard imaging modalities.

**Recent Findings:**

Although MRU is still largely performed in major academic or specialty imaging centers, more and more applications in the pediatric setting have been described in the literature.

**Summary:**

MRU is a comprehensive imaging modality for evaluating multiple pediatric urologic conditions combining excellent anatomic detail with functional information previously only available via renal scintigraphy. While generally still reserved for problem solving, MRU should be considered for some conditions as an early imaging technique.

## Introduction

There are many clinical indications to image the urinary tract in the pediatric population. Urinary tract dilatation (UTD), detected pre- or post-natally, is one of the most common reasons to image the urinary tract. Magnetic resonance urography (MRU) is increasingly being used for comprehensive anatomic and functional evaluation of the urinary tract in children. MRU has been in clinical development in children since the early 2000s and has been subsequently refined and improved over time. It is now routinely used in clinical care in many institutions over the last 5 to 10 years [1, 2]. The information that can be provided with MRU is similar to that acquired with a combination of ultrasound, computed tomography (CT), excreted urography, and renal scintigraphy and it does so with no exposure to ionizing radiation.

## Common Imaging Modalities for Pediatric Urologic Conditions

Ultrasonography (US) is the most commonly employed imaging modality to evaluate the kidneys and bladder pre- and post-natally. US has the advantages of being performed without sedation or ionizing radiation and is non-invasive. US generally provides sufficient anatomical detail of renal anatomy and any parenchymal changes (diffuse thinning, altered echogenicity, cysts, etc.) and is the primary imaging modality used to identify and grade hydronephrosis. However, ultrasound is limited for visualization of the ureters, especially when non-dilated, and is particularly limited at the levels of the mid-ureter and ureterovesical junction. On the other hand, when there is marked ureterectasis, it also can be difficult to fully characterize urinary tract anatomy by US due to anatomic distortion and the relatively limited field of view. Furthermore, US provides no information about renal function; although, speculatively US performed with an intravascular contrast material (i.e., microbubble contrast) may provide some information regarding differential perfusion in the future avoiding both nuclear medicine and MRI-based contrast agents. US technique can be affected by many patient-specific parameters such as bowel gas, body habitus (e.g., scoliosis and obesity), and patient cooperation.

Voiding cystourethrography (VCUG) is another commonly employed imaging modality for urologic conditions in the pediatric population and is most often used for diagnosing vesicoureteral reflux and assessing the morphology of the bladder and urethra. VCUG requires placement of a urethral catheter and uses intermittent, low-dose fluoroscopy to image contrast material instilled into the urinary tract. In the absence of vesicoureteral reflux, no information is gained regarding the upper tract collecting system, and VCUG does not provide information about the renal parenchyma.

Scintigraphic studies can provide a range of information about the urinary tract depending on the radiopharmaceutical employed. Diuretic renal scintigraphy using mercaptoacetyltriglycine (MAG3) provides functional (i.e., differential renal function based on plasma flow) and drainage information. Renal cortical scintigraphy using dimercaptosuccinic acid (DMSA) provides information about the renal parenchyma (i.e., differential renal function based on cortical binding and detection of focal scarring), while diethylenetriaminepentaacetic acid (DTPA) provides information about renal functional (i.e., differential renal function based of glomerular filtration) and drainage. The anatomic detail provided by scintigraphy is inherently limited, but the functional information provided remains the imaging reference standard. Scintigraphic studies necessarily expose patients to ionizing radiation but only rarely require sedation.

CT can be useful for some pediatric urologic conditions but is typically only used as a first-line imaging modality for renal masses and urinary tract calculi in the pediatric population. In part, this is due to the fact that CT necessitates exposure to ionizing radiation. CT urography (CTU) is relatively commonly employed in the adult population but is infrequently used in pediatrics because it generally requires multiple image acquisitions (non-contrast, parenchymal or nephrographic phase, ureteral or excretory phase). The number of image acquisitions can be decreased using dual-energy CT which provides a virtual non-contrast imaging series or by performing “split-bolus” CTU, where two separate administrations of intravenous contrast material allow nephrographic and excretory phase information to be obtained from the same image acquisition [3]. CT can provide a qualitative assessment of renal function if multiple phases are acquired, but this is typically impractical and comes at a cost of radiation dose.

## Basics of MRU Technique

Pediatric MRU can be performed at 1.5 or 3 Tesla (T) in children of any age. 3 T magnets generally offer superior spatial resolution, which is helpful particularly in younger children, with improved visualization of small urinary tract structures. However, 1.5 T magnets generally allow for more homogeneous fat saturation and are less susceptible to artifacts, such as dielectric effect, T2* effects of excreted gadolinium, and any artifacts from surgical material. Imaging is performed with multi-element phased-array surface coils.

MR urography can refer to anatomic imaging of the kidneys and collecting system but more commonly refers to anatomic imaging in combination with functional imaging, the latter of which requires administration of intravenous gadolinium-based contrast material. Anatomic imaging of the abdomen and pelvis is performed, including sequences that focus on the renal parenchyma (T1- and T2-weighted sequences) and sequences focused on the urinary tracts. Sequences targeted at the urinary tract include high-resolution 2D and 3D T2-weighted images, which when obtained in a 3D fashion allow multiplanar reformatting and can be used to make a variety of reconstructions (e.g., volume-rendered and maximum intensity projection images).

Functional MR urography allows the determination of differential renal function and allows assessment of renal excretion into the collecting systems. Functional imaging is obtained dynamically over a 10- to 15-min period of time following administration of intravenous gadolinium-based contrast material. By imaging multiple times over 15 min, renal parenchymal contrast uptake and excretion are visualized and later quantified with post-processing techniques. This allows the measurement of differential renal function (based on renal volumes or glomerular filtration) and time vs. signal intensity washout/excretion curves. Detailed reviews of these calculations have been described [4•]. The provided data is comparable to that obtained by scintigraphic studies; however, scintigraphy remains an accurate and reliable modality for cases that do not require the additional anatomic information provided by MRU. Newer MRI techniques likely will be forthcoming to more specifically non-invasively evaluate the renal parenchyma for findings of inflammation and fibrosis [5–8].

While MRU has the advantages of being a radiation-free imaging modality and providing the greatest anatomic detail of any modality for imaging the urinary tract, it does have some limitations that need to be considered. First, MRU exams require the patient to lie still in the bore of the magnet for up to 60 to 90 min. Some children can achieve this without difficulty, particularly if distraction techniques (video goggles, etc.) are employed, but others will require sedation/anesthesia or anxiolysis to complete their exam. Second, administration of intravenous gadolinium-based contrast material used to be considered entirely benign but is being increasingly scrutinized due to evidence of retention of gadolinium in the body [9].

## How the MRU Works in Practice

Generally children younger than 8–10 years of age or those with developmental delay will require some form of anesthesia or sedation to prevent motion artifacts, which is a relative disadvantage compared to other imaging techniques. The age at which children require sedation also will depend on prior experience and tolerance of bladder catheterization, which is a necessary part of the exam.

Patients are instructed to arrive 60–90 min prior the appointment time for exam preparation. If performed without sedation, patients are instructed to remain NPO for 4 h. If sedated, NPO guidelines are determined by the sedation team. A bladder catheter is placed, which allows continuous drainage of urine to prevent patient discomfort and facilitate excretion and identification of the urethra on imaging. A peripheral IV catheter is placed for administration of hydration, diuretic (typically furosemide), and IV contrast material. If needed, a separate IV is placed for sedation purposes. Initially, the bladder catheter is clamped to allow identification and assessment of the bladder. Thereafter, the catheter is left to drain. At the conclusion of the exam, the catheter is removed unless it is needed for other testing or procedures. The total MR scanning time is 45 to 90 min, depending on the exact protocol and degree of patient cooperation. Thus, the overall total time for the procedure is approximately 2 to 3 h (Table 1).

**Table 1:** Our pediatric MRU protocol

Patient arrives 60–90 prior to exam time	NPO 4 h if non-sedate (otherwise set by anesthesia)
Place bladder catheter and clamp	
Place IV catheter (two IV catheters if sedation is needed)	
IV hydration	10 mL/kg IV saline over 15 min for sedated patients or 30 min for non-sedated
T2-weighted single-shot fast spin-echo without/with fat suppression	Sagittal, coronal
Unclamp bladder catheter	
IV diuretic	0.5 to 1 mg/kg (max dose = 40 mg)
T2-weighted fast spin-echo with fat suppression	Axial
High spatial resolution 3D T2-weighted fast spin-echo without and with fat suppression	Coronal
3D T1-weighted gradient recalled echo with fat suppression	Coronal
IV Dotarem	0.2 mL/kg at 0.2 mL/s
3D T1-weighted gradient recalled echo with fat suppression	Coronal, 15 min dynamic post-contrast
3D T1-weighted gradient recalled echo with fat suppression	Sagittal, coronal, axial
Remove IV and bladder catheter	

## Common Indications for MRU

Common indications for pediatric MRU include evaluation of complex renal and upper urinary tract anatomy, suspected urinary tract obstruction, operative planning, post-operative complications, and functional assessment. Generally, patients have already undergone conventional imaging tests such as US, VCUG, and/or renal scintigraphy and yet the clinician still needs additional information for management. As such, MRU is typically employed as a problem solving or surgical planning modality. MRU can delineate anatomy in the presence or absence of collecting system dilation, which can be a limiting factor in US evaluation. Additionally, MRU can visualize the entire course of the ureter and identify ectopic insertions as well as sites and potential causes of narrowing or obstruction, including identification of crossing vessels as a cause of ureteropelvic junction obstruction. In planning for surgery or evaluating post-surgical changes, MRU can provide detailed anatomic assessment for the surgeon with the ability to make 3D reconstructions of the entire renal and upper urinary tract. MRU functional assessment can provide quantitative data of differential renal function.

## Common Clinical Applications

### Collecting System Abnormalities

One of the most common abnormalities detected by prenatal ultrasound is UTD, occurring in 1–2% of all pregnancies [10–12]. Most often, this finding is transient and resolves early in life. However, multiple clinically relevant abnormalities initially present in this fashion and require further evaluation or intervention to prevent complications such as urinary tract infection (UTI), urinary stone formation, and renal dysfunction/injury [10, 11, 13–15]. Of note, it has recently been recognized that children with congenital obstructive nephropathy will go on to develop end-stage renal disease in adulthood at a higher rate than previously expected, with need for renal replacement therapy not manifesting until the fourth decade of life [13, 15]. Following transient/physiologic UTD, the most common etiologies of UTD include ureteropelvic junction (UPJ) obstruction, vesicoureteral reflux, ureterovesical junction obstruction (megaureter), multicystic dysplastic kidney disease (MCDK), and posterior urethral valves [10, 14].

MRU can be used in the assessment and characterization of the majority of causes of UTD and obstruction with the exception of vesicoureteral reflux and probably posterior urethral valves, which are better assessed with VCUG as described above. The added value of MRU to the traditional imaging modalities of US, VCUG, and renal scintigraphy in the setting of dilated collecting systems largely relates to accurate anatomic descriptions, parenchymal evaluation, and functional assessment. Many of the common causes of collecting system dilation have one or more congenital anatomical abnormalities that can go undetected on traditional imaging modalities. Additionally, MRI is superior to US and renal scintigraphy for evaluating the renal parenchyma for inflammation, scarring, and cortical thinning, which are frequently associated with the various causes of urinary tract dilation. Further, the functional information provided by MRU can be helpful for surgical planning (e.g., guiding the decision for heminephrectomy vs. ureteral reimplantation) and prognosticating.

### Ureteropelvic Junction Obstruction

**Fig. 1:**
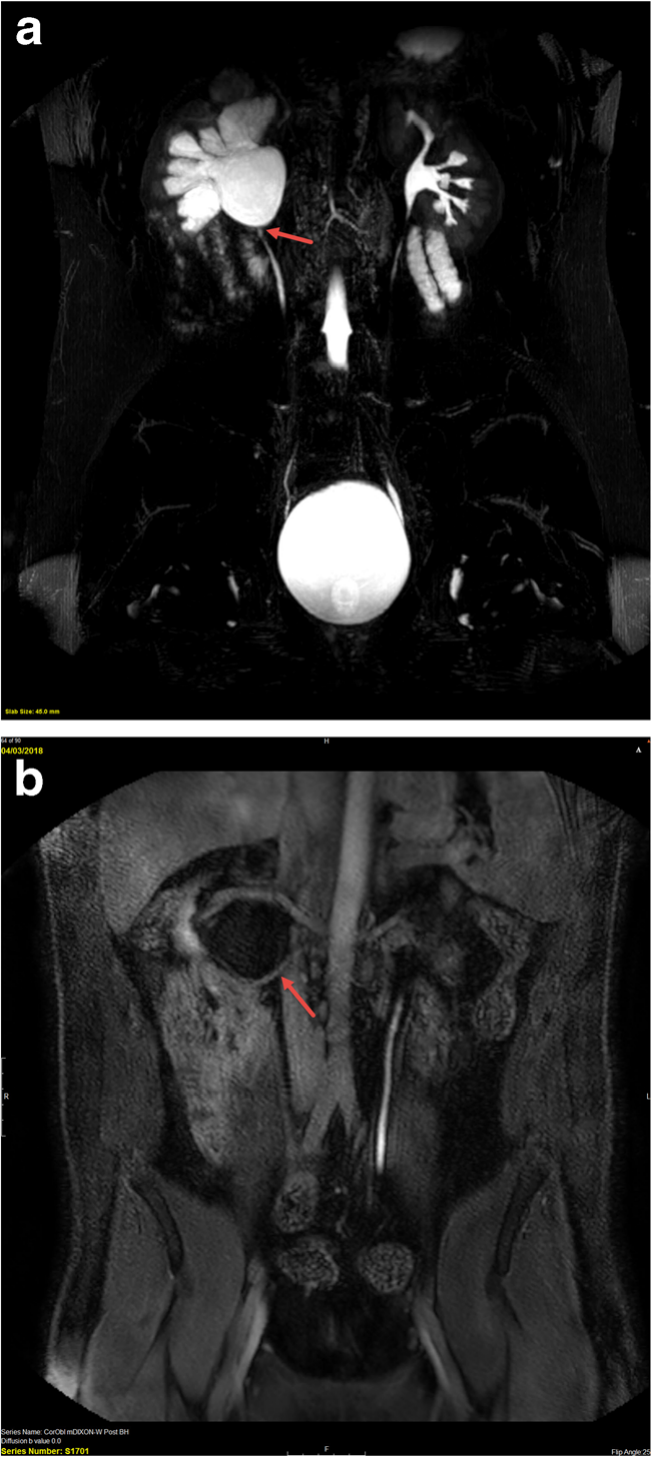
Sixteen-year-old with right flank pain and right hydronephrosis discovered on renal US. **a** 3D T2-weighted image shows moderate right pelvocaliectasis with an abrupt transition to a normal caliber ureter (arrow) consistent with a ureteropelvic junction obstruction. **b** Post-contrast excretory phase image shows a crossing accessory renal artery (arrow) supplying the lower pole of the right kidney. The renal pelvis is dilated proximal to the crossing vessel, and the ureter distal to it is normal in caliber. There is no visible excreted contrast material in the right renal collecting system, while contrast material is seen in a normal caliber left ureter

UPJ obstruction is most commonly due an intrinsic abnormality of the proximal ureter and is the most common cause of upper urinary tract obstruction [10, 11, 13, 16, 17]. Generally, this is a unilateral condition and is initially diagnosed by ultrasound. Preservation of functional renal mass is the primary therapeutic goal by resolving the obstruction, usually surgically [10, 13, 15]. MRU allows for the diagnosis of UPJ obstruction, the identification of any extrinsic cause of the obstruction (e.g., crossing vessel) that would change surgical approach, and the ability to detect asymmetric split renal function and parenchymal alterations, which are important indications for surgery (Fig. 1) [16–18].

### Mid-Ureteral Stricture

**Fig. 2:**
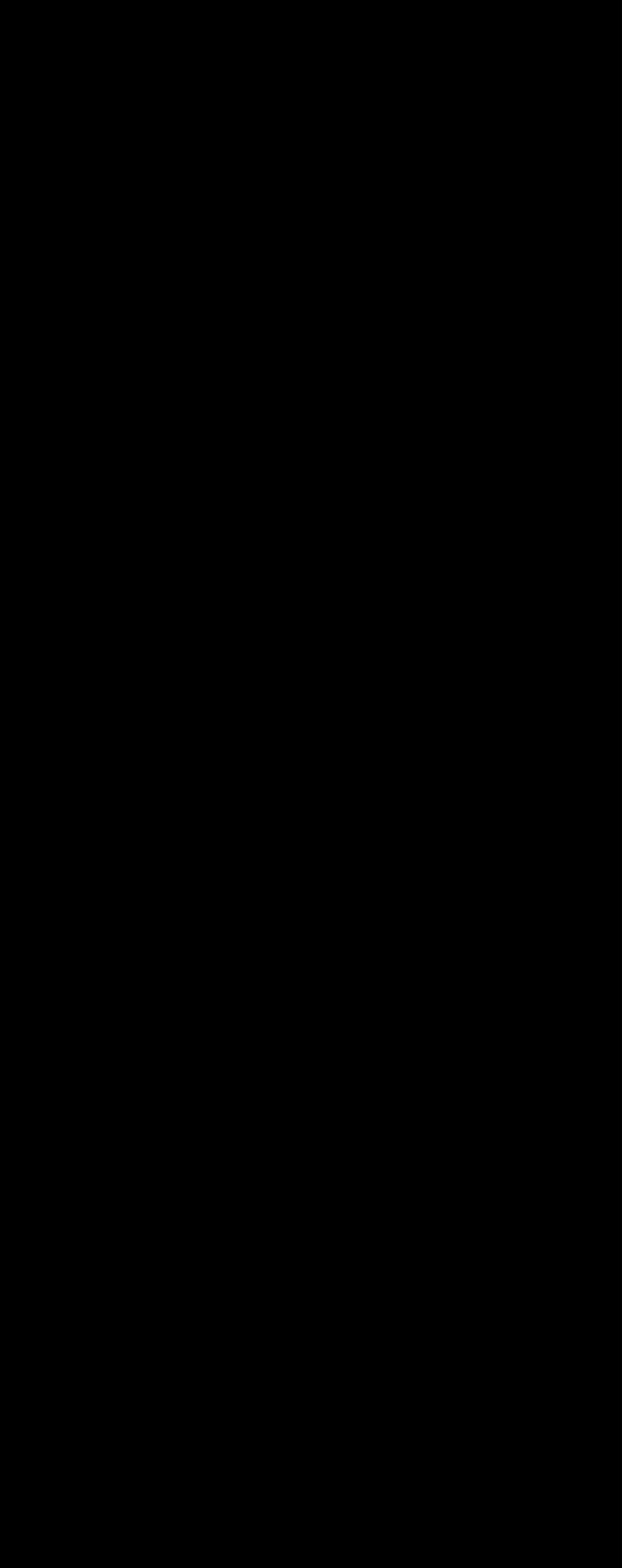
Five-month-old female with antenatal diagnosis of hydronephrosis with atypical findings on US. **a** Coronal SSFE non-fat saturated image demonstrates abrupt cutoff of the mid-ureter. No distal ureter could be seen on additional images. **b** Antegrade nephrostogram confirmed the mid-ureteral stricture

Congenital mid-ureteral stricture is a rare cause of urinary tract dilation (Fig. 2) [12, 19–21]. This is a difficult diagnosis to make with US, renal scintigraphy, and VCUG and can be misdiagnosed as UPJ or ureterovesical junction (UVJ) obstruction/primary megaureter [19, 20, 22]. In a study of 26 children, Arlen et al. showed that children with mid-ureteral strictures underwent a mean of 2.7 imaging studies with less than half (42%) receiving the correct diagnosis prior to MRI, which lead to a definite diagnosis in all cases [20]. In the same paper, the authors found that these strictures are commonly associated with additional renal anomalies which can be diagnosed and characterized with MRU including contralateral mid-ureteral stricture, MCDK, collecting system duplication, paraureteral diverticulum, and ectopic ureterocele [20, 23]. In addition to strictures, additional rare causes for hydroureteronephrosis include congenital ureteral valves [24, 25] and ureteral fibroepithelial polyps [26], both of which can occur at various levels of the ureter and can be diagnosed with MRU, enhancing the surgical plan with this knowledge.

### Ureterovesical Junction Obstruction and Congenital Primary Megaureter

**Fig. 3:**
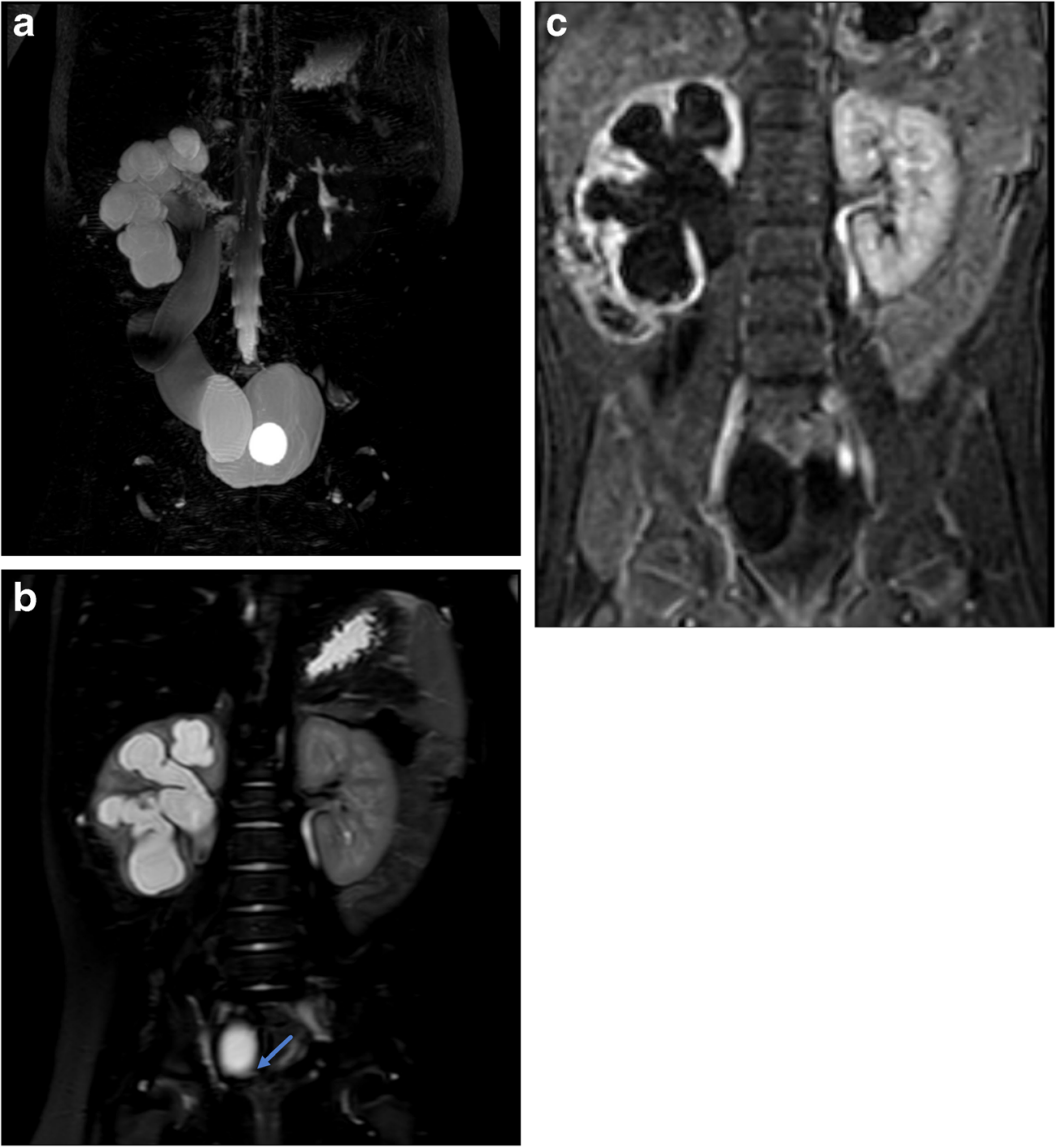
Six-month-old with history of febrile UTI. Initial ultrasound showed moderate to severe right hydronephrosis and megaureter. VCUG showed grade 3 left-sided reflux, no right reflux, and bilateral periureteral diverticula. Together with the MRU findings, this patient was diagnosed with refluxing, obstructive congenital primary megaureter. **a** Coronal maximum intensity projection (MIP) image from a 3D T2-weighted sequence shows marked right pelvocaliectasis and a dilated, tortuous right ureter. A bladder catheter is in place with a fluid-filled balloon in the bladder lumen. **b** Coronal T2-weighted single-shot fat suppressed image shows abrupt narrowing and “beaking” of the distal ureter at the ureterovesical junction consistent with obstruction. **c** Single image from the dynamic post-contrast sequence shows diffuse parenchymal thinning of the right kidney with delayed excretion into the renal collecting system relative to the left, indicative of clinically relevant obstruction

Primary UVJ obstruction and obstructive congenital primary megaureter are due to obstruction of the ureter as it enters the bladder or dysfunctional or absent peristalsis of the distal ureter leading to variable degrees of upper urinary tract obstruction [27–29]. Often hydroureteronephrosis is diagnosed on prenatal ultrasound with a retrovesical ureteral measurement of ≥ 7–10 mm [28, 29]. All infants with prenatal ureteral dilation should receive follow-up imaging with postnatal US, and, if persistent, they should also undergo VCUG to exclude reflux or urethral valves as a cause of dilation. If both are excluded, then children typically undergo renal scintigraphy to confirm obstruction at the UVJ. A large number of children experience spontaneous resolution of congenital primary megaureter by 5 years of age (73–92%) [29–31]. However, there is significant variability and some children will require surgical reimplantation to prevent complications leading to irreversible renal injury and decreased renal function. MRU can identify and provide detailed anatomic information regarding the narrowed segment of distal ureter, while also allowing detailed evaluation of the kidneys and collecting systems and urinary tract drainage. Associated abnormalities such as concomitant UPJ obstruction can be seen. Renal parenchymal thinning or scarring can be seen as a result of urinary tract infections, which are increased in prevalence with UVJ obstruction [30]. Differential renal function can be a predictor of the need for surgery [28, 29] and can be evaluated with MRU (Fig. 3).

### Renal Ectopia/Fusion Anomalies

**Fig. 4:**
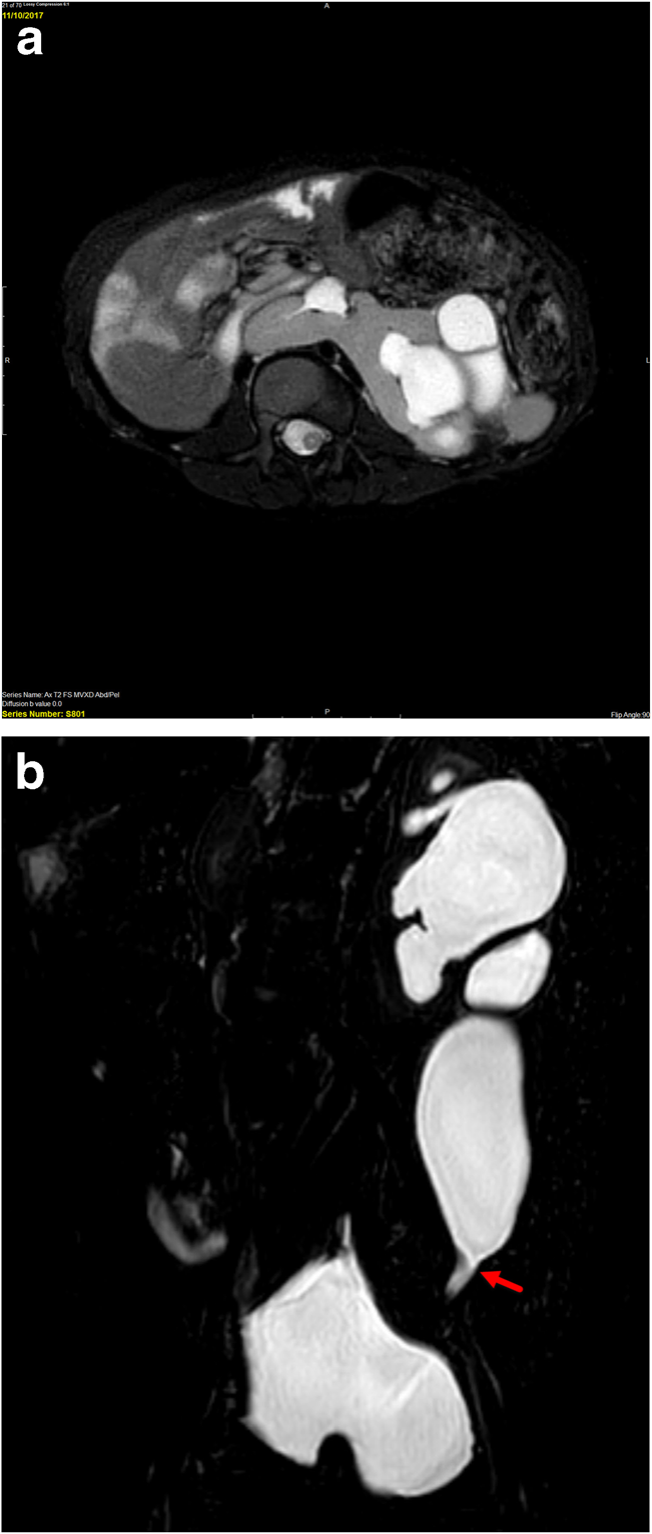
Four-year-old with history of horseshoe kidney who has previously undergone left pyeloplasty but has persistent hydronephrosis and ureteral dilation and clinical concern for obstruction. **a** Axial T2-weighted fat suppressed image shows renal parenchyma crossing the midline anterior to the spine (blue arrow) consistent with a horseshoe kidney. There is moderate pelvocaliectasis of the left moiety (red arrow). **b** Coronal T2-weighted image shows abrupt narrowing of the distal left ureter (red arrow). At surgery, the stricture was found to be related to scar tissue from prior pyeloplasty

Ectopia and fusion anomalies represent a spectrum of anomalies related to abnormal embryonic migration with various degrees of failure of ascent of the developing kidney [32, 33]. Some of the commonest anomalies in this spectrum include pelvic kidneys, cross fused renal ectopia, and horseshoe kidneys. Pelvic kidneys are simply those which fail to ascend superior to the pelvis. Cross fused ectopic kidneys describe a scenario in which one kidney fails to ascend, crosses midline, and commonly fuses with the lower pole of the contralateral kidney. Horseshoe kidneys are those in which there is a rotational anomaly with fusion of the medial aspects of the lower poles of both kidneys. Horseshoe kidneys are typically located more inferiorly than normal kidneys with the isthmus anterior to the aorta and inferior vena cava (IVC) at the L3 level or below and below the inferior mesenteric artery [32, 34]. Renal fusion anomalies, including crossed renal ectopia and horseshoe kidney, are at increased risk for complications including urinary tract obstruction (e.g., UPJ obstruction), infection, urolithiasis, and rarely tumor [34, 35]. US is typically the initial imaging study of choice for diagnosing fusion anomalies and many are identified incidentally. Renal scintigraphy can also be useful to identify the congenital anomaly, assess renal parenchymal mass and split function, and assess collecting system drainage [36]. MRU provides greater anatomic detail of both the parenchyma and collecting system than either of these modalities and can provide functional assessment of the collecting system for abnormalities, such as UPJ obstruction (vs. non-obstructive collecting system ectasia which is common in these anomalies) which are increased in the setting of fusion anomalies (Fig. 4) [35]. MRU also may allow more precise segmentation of the fused renal moieties to provide differential function.

### Ectopic Ureter

**Fig. 5:**
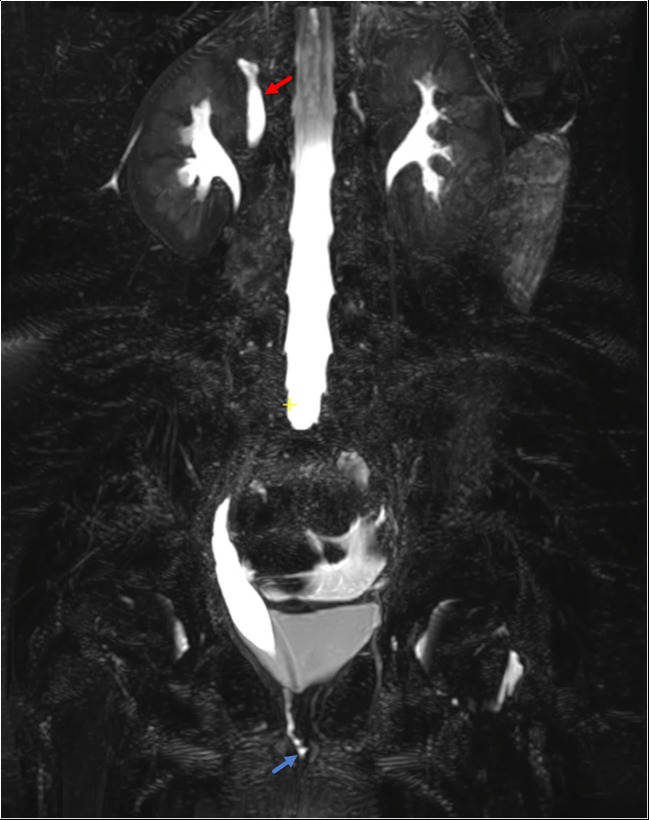
Six-year-old female with history of continuous urinary incontinence. Coronal 3D T2-weighted image demonstrates dilation of the single calyx right upper pole moiety (red arrow). The distal ureter is mildly dilated and demonstrates tapering at the level of the perineum and ultimately is seen draining at the level of the introitus (blue arrow)

Mostly commonly occurring in the setting of duplex collecting systems, ectopic ureters can be challenging to diagnose and their insertions can be difficult to identify with conventional imaging methods. In girls, ectopic ureters that insert into the vagina or into the urethra below the level of the external sphincter mechanism can result in continuous urinary incontinence in an otherwise continent child (Fig. 5). In boys, the ectopic ureter can insert into the posterior urethra at the level of the sphincter or elsewhere in the genital tract. Clinically, male children can present with recurrent urinary tract infections or pelvic pain [4]. MRU has been demonstrated to have high accuracy for depicting ectopic ureters with the addition of a single 3D T2-weighted fast spin-echo (non-contrast) sequence that provides high spatial resolution [37••, 38, 39, 40]. MRU allows for assessment of parenchymal quality, volume, and function thus guiding the decision to remove the kidney (e.g., upper moiety nephrectomy) vs. perform ureteroureterostomy. This additional information is critical in allowing the urological team to determine the optimal treatment plan and counsel the patient and their family with the aid of images about the nature of the planned surgery, route of the surgery, i.e., pelvic, transperitoneal, or retroperitoneal, and also the modality to be used for the actual surgery (i.e., open surgery vs. robotic-assisted laparoscopic surgery). Ultimately, this guides the surgeons in delivering the most appropriate care and ensures the best clinical outcome for each individual patient. In contrast to other urologic abnormalities, MRU should be considered the primary imaging modality for suspected ectopic ureter.

### Post-Surgical

**Fig. 6:**
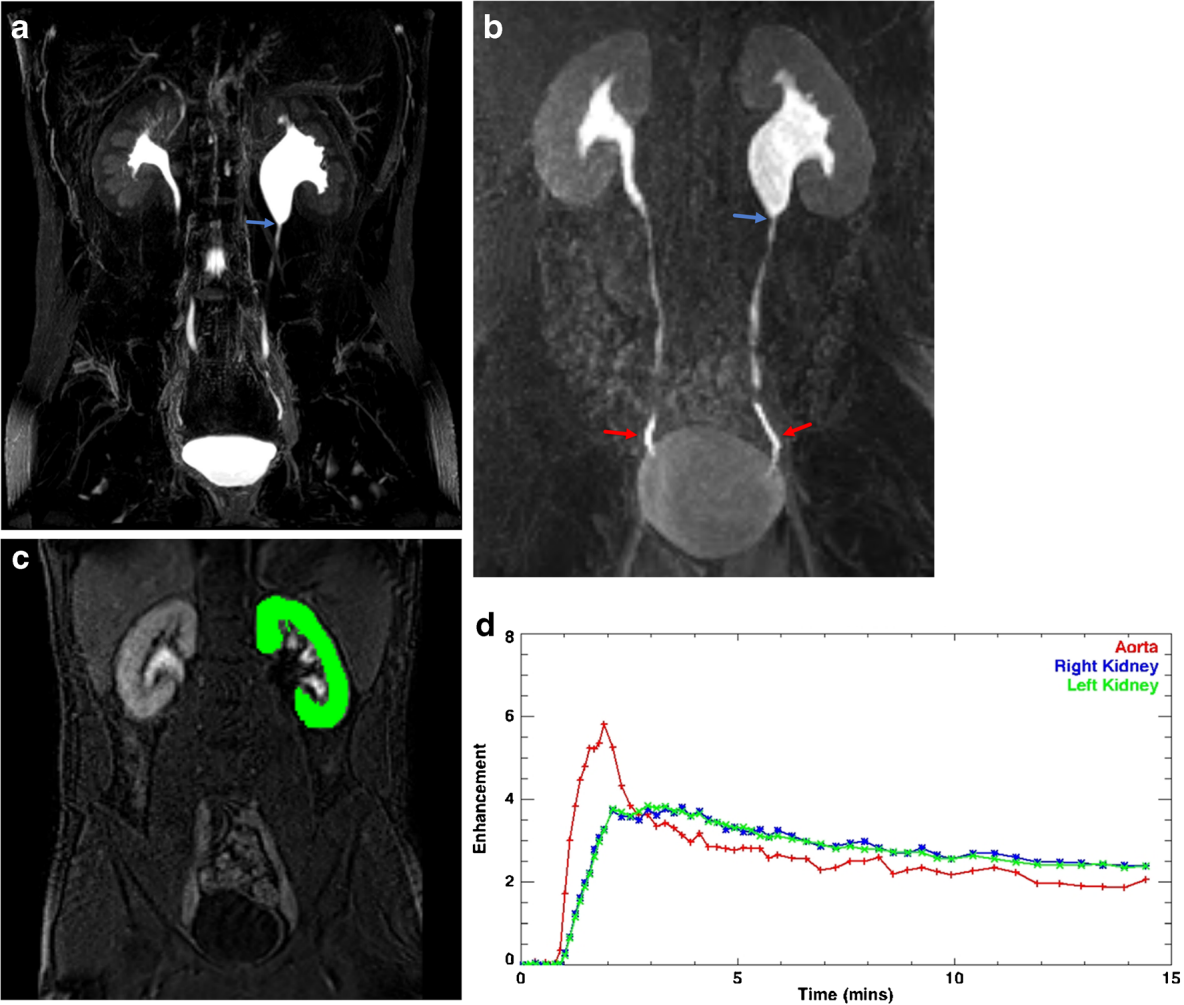
Seventeen-year-old with left hydronephrosis and cyclic vomiting who has previously undergone left pyeloplasty. **a** Maximum intensity projection (MIP) reconstruction of 3D T2-weighted fast spin-echo image showing abrupt narrowing of the left renal pelvis at the level of the ureteropelvic junction (arrow). **b** MIP reconstruction of a post-contrast excretory image shows symmetric renal enhancement and excretion of contrast. Contrast readily flows past the narrowed, yet patent UPJ (blue arrow) as evidenced by the presence of contrast within both distal ureters (red arrows). On dynamic imaging, there was symmetric passage of contrast material through the kidneys and into the renal collecting systems and ureters suggesting no clinically relevant obstruction. **c** Example of region of interest overlying the left kidney performed during segmentation. **d** Time-vs.-signal intensity curves from the kidneys and abdominal aorta are obtained from dynamic post-contrast MR urograms post-processing; in this case, the curves demonstrate symmetric renal uptake and excretion confirming lack of obstruction in the left kidney

MRU can be helpful in the post-operative setting in several scenarios. Following pyeloplasty for UPJ obstruction, MRU allows assessment for reduction in the degree of hydronephrosis as well as improvement in split function and collecting system drainage (Fig. 6). This was shown in a paper by Kirsch et al. demonstrating that in a study of 24 patients, more than 90% of the children showed improvement in the differential renal function and estimated GFR following surgery [41]. In the children that did not improve, this allowed decision making regarding stent placement vs. observation on the basis of renal transit time and degree of persistent hydronephrosis. Further, MRU depicts the reconstructed UPJ in exquisite anatomic detail allowing evaluation of caliber and assessment for residual anatomic narrowing or restenosis.

**Fig. 7:**
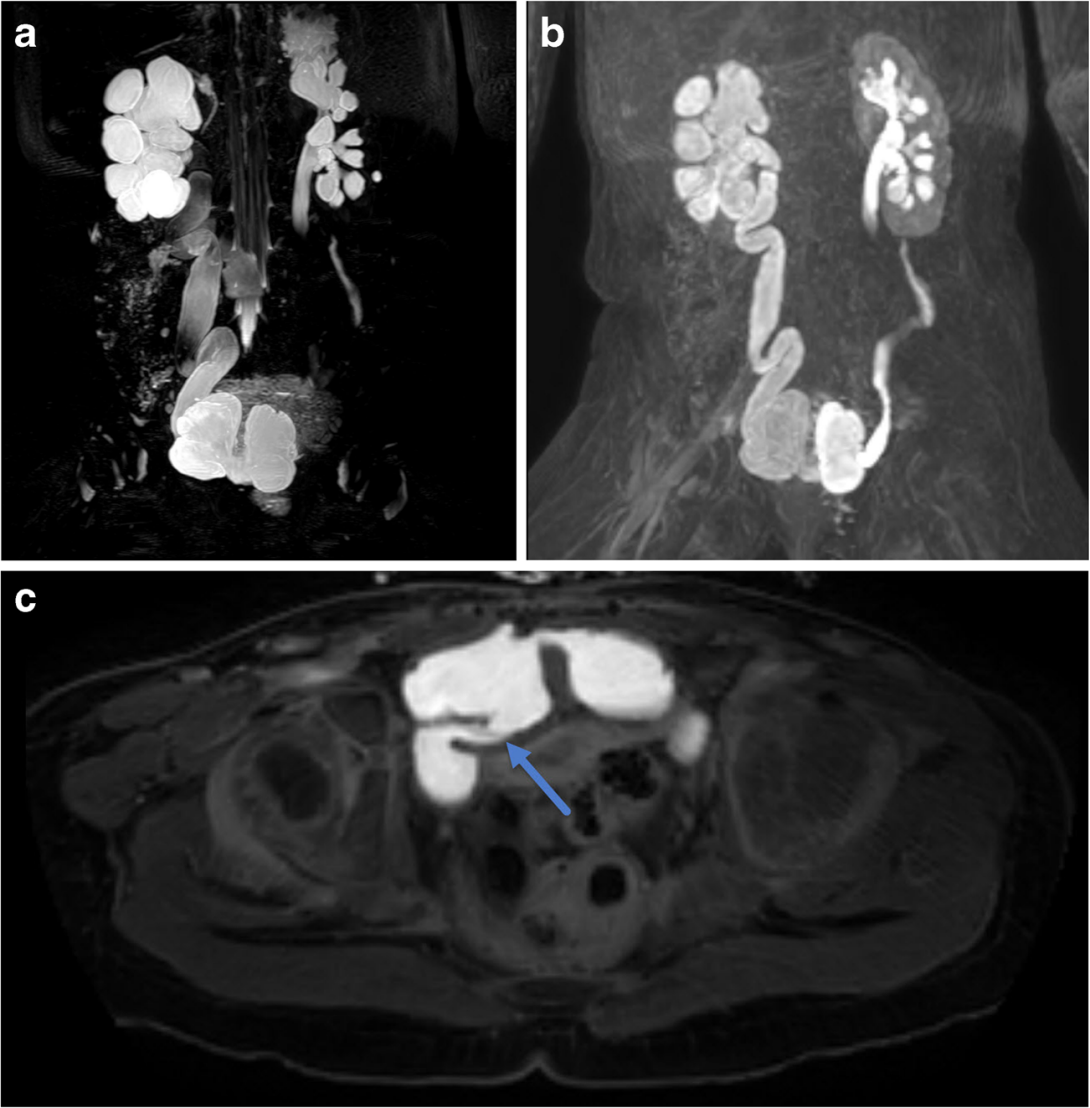
Three-year-old born premature at 27 weeks gestational age with history of bladder exstrophy post-repair with neobladder creation and vesicostomy. **a** MIP reconstruction of 3D T2-weighted fast spin-echo image shows marked right pelvocaliectasis and a dilated, tortuous right ureter. There is mild left hydroureteronephrosis. The neobladder has a bilobed appearance. **b** MIP reconstruction of 3D post-contrast excretory phase imaging shows symmetric excretion of contrast into both renal collection systems and ureters which freely flows into the bilobed neobladder. **c** Axial post-contrast excretory phase image shows a patent right ureterovesical junction (arrow)

Another use of MRU is in the post-surgical setting of complex anatomy. In a patient with persistent hydroureter in the setting of bladder reconstruction, MRU shows excellent anatomic detail of ureterovescial junction, allowing assessment for intrinsic and extrinsic obstruction and assessment of drainage (Fig. 7).

### Renal Transplant

**Fig. 8:**
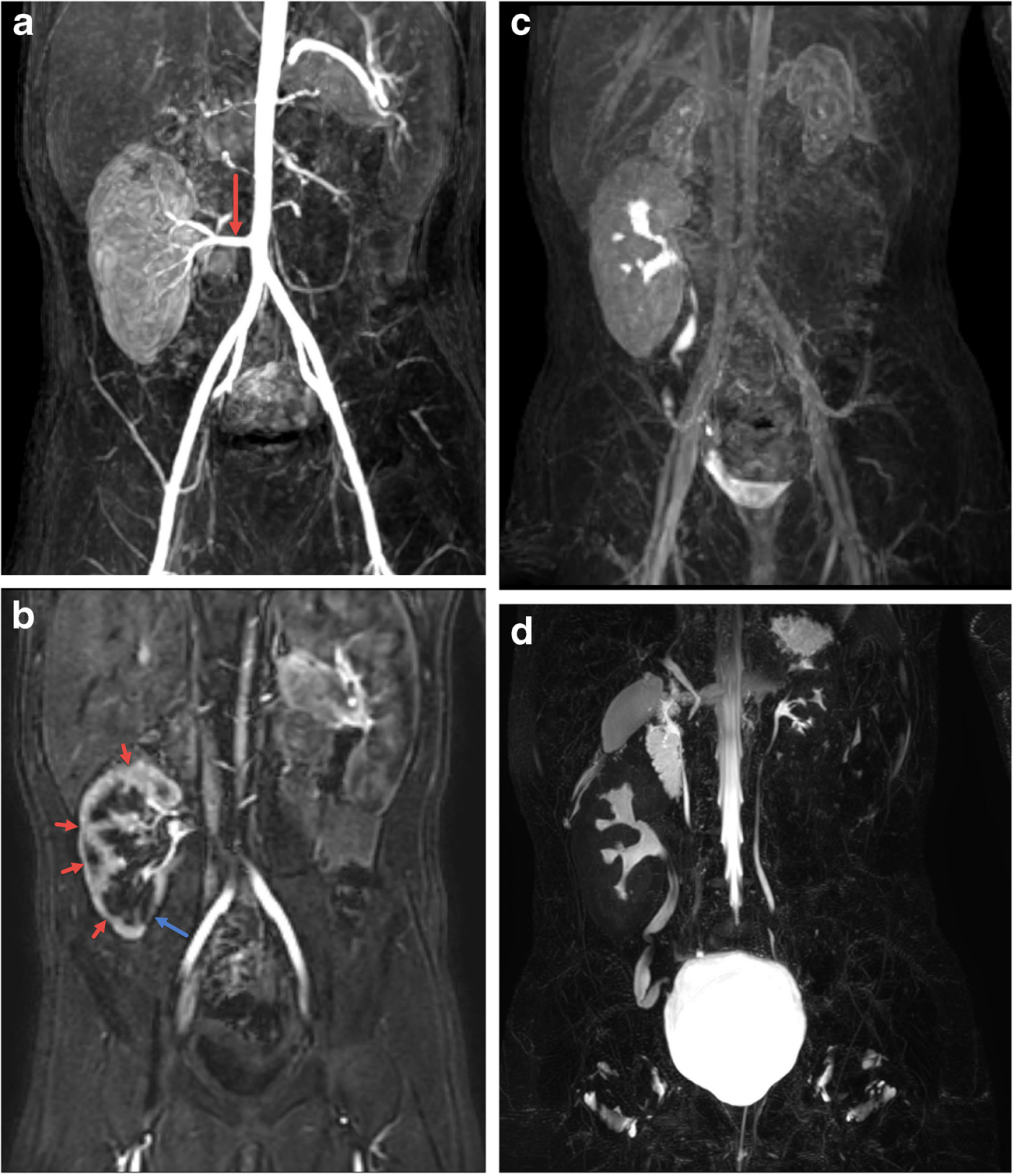
Nine-year-old with history of hemolytic uremic syndrome who has undergone right lower quadrant renal transplant. **a** Maximum intensity projection (MIP) reformat image from the arterial phase of the dynamic post-contrast sequence shows a patent artery supplying the right lower quadrant renal transplant (arrow). **b** Subsequent MIP image from the corticomedullary phase shows multifocal regions of parenchymal thinning and scarring (red arrows) with the most pronounced region of scarring in the lower pole medially (blue arrow). **c** MIP delayed post-contrast excretory phase image shows excretion of contrast into the renal transplant collecting system and ureter without dilation and with free passage of contrast into the bladder. The small native kidneys can also be seen. **d** MIP reformat of 3D T2-weighted fast spin-echo image shows no pelvocaliectasis with mild tortuosity of the ureter. The small native kidneys and ureters can also be seen

Evaluation for complications related to renal transplantation is a relatively common indication for imaging in children. Typically, evaluation in the immediate post-operative setting is performed with US including Doppler for evaluating the vasculature. Medium and long-term imaging follow-up is also generally by US in conjunction with percutaneous biopsy for suspected rejection. MRI has a potential role in evaluating renal transplants non-invasively but is largely reserved for problem solving [42–44]. MRI can be used to assess peri-transplant fluid collections and to evaluate both the supplying vessels and perfusion of the allograft (dynamic post-contrast). Functional MRU can be used to assess drainage of the transplant collecting system. MRI can also assess the renal parenchyma for signs of inflammation such as edema or more long-term damage, including parenchymal scarring and thinning (Fig. 8).

## Conclusion

MRU provides probably the most complete assessment of the urinary tract in children, allowing detailed evaluation of the renal parenchyma, collecting systems and ureters, and the bladder and providing both static and dynamic functional information. As such, MRU has the potential to be contributory to the evaluation of a wide variety of pediatric urologic abnormalities. Currently, MRU is typically reserved for problem solving after traditional imaging modalities of US, VCUG, and/or renal scintigraphy have not provided all the necessary information for clinical decision making. In the future, the use of MRU may increase in the evaluation of children with genitourinary anomalies, particularly in complex patients in which urologists have specific questions to be answered.
